# Identifying discriminative features of brain network for prediction of Alzheimer’s disease using graph theory and machine learning

**DOI:** 10.3389/fninf.2024.1384720

**Published:** 2024-06-18

**Authors:** S. M. Shayez Karim, Md Shah Fahad, R. S. Rathore

**Affiliations:** ^1^Department of Bioinformatics, Central University of South Bihar, Bihar, India; ^2^Department of Computer Science and Engineering, Birla Institute of Technology, Ranchi, India

**Keywords:** machine learning, Alzheimer’s disease, connectome, neuronal connections, brain regions, fMRI, graph theory, network parameters

## Abstract

Alzheimer’s disease (AD) is a challenging neurodegenerative condition, necessitating early diagnosis and intervention. This research leverages machine learning (ML) and graph theory metrics, derived from resting-state functional magnetic resonance imaging (rs-fMRI) data to predict AD. Using Southwest University Adult Lifespan Dataset (SALD, age 21–76 years) and the Open Access Series of Imaging Studies (OASIS, age 64–95 years) dataset, containing 112 participants, various ML models were developed for the purpose of AD prediction. The study identifies key features for a comprehensive understanding of brain network topology and functional connectivity in AD. Through a 5-fold cross-validation, all models demonstrate substantial predictive capabilities (accuracy in 82–92% range), with the support vector machine model standing out as the best having an accuracy of 92%. Present study suggests that top 13 regions, identified based on most important discriminating features, have lost significant connections with thalamus. The functional connection strengths were consistently declined for substantia nigra, pars reticulata, substantia nigra, pars compacta, and nucleus accumbens among AD subjects as compared to healthy adults and aging individuals. The present finding corroborate with the earlier studies, employing various neuroimagining techniques. This research signifies the translational potential of a comprehensive approach integrating ML, graph theory and rs-fMRI analysis in AD prediction, offering potential biomarker for more accurate diagnostics and early prediction of AD.

## 1 Introduction

Alzheimer’s disease (AD) is a progressive neurological condition that affects millions of individuals worldwide (Li et al., 2022). It is distinguished by cognitive decline, memory loss, and behavioral abnormalities. One of the main risk factors for developing neurodegenerative diseases is aging, as it leads to various cellular and molecular changes that impair the brain’s ability to cope with stress and damage (Mayne et al., 2020). The diagnosis of AD is usually based on clinical criteria, neuropsychological tests, and biomarkers such as cerebrospinal fluid (CSF) and amyloid PET imaging (Cummings, 2012; National Institute on Aging, 2023). However, these methods are invasive, expensive, and not widely available. The growing burden of mild cognitive impairment (MCI), a prodromal stage of AD and dementia (Sperling et al., 2011) is a major challenge for health care systems around the world. Primary care physicians and specialists will need to be prepared to diagnose and manage them in an increasingly aging population (Livingston et al., 2020). Therefore, there is a need for alternative and non-invasive methods to diagnose AD at an early stage (Alroobaea et al., 2021).

Cognitive dysfunction is the main symptom of AD, which is diagnosed mainly by structural brain changes. However, functional connectivity, which indicates the functional activity synchronization between distant brain regions, may change even before structural alterations. The identification of biomarkers can help clinicians detect AD early on, and initiate treatment. Resting-state functional magnetic resonance imaging (rs-fMRI) has emerged as a powerful tool for investigating neurological disorders. By analyzing functional connectivity (FC) networks derived from rs-fMRI data, we can quantify the functional interactions between brain regions, providing valuable insights for diagnosis. Toward this aim, many studies have investigated the resting-state networks (RSN) among MCI and AD individuals (Badhwar et al., 2017; Farràs-Permanyer et al., 2019). RSNs are spatially coherent, blood-oxygen-level-dependent (BOLD) signals detected in fMRI. They are made up of regional patterns commonly involved in brain functions such as sensory, attention, default mode processing etc. Many of these studies have revealed altered FC and disruptions in RSNs, primarily of default mode network (DMN) and fronto-pariatal network (FPN), which can serve as early biomarkers for predicting the progression to AD (Sorg et al., 2007; Zhukovsky et al., 2023). In addition, altered (increased or decreased) FC patterns have been reported in anterior and posterior cingulate cortex (ACC and PCC) regions. It is argued that this could also serve as biomarker (Dickerson and Sperling, 2008; Yuan et al., 2022). These findings support the potential of fMRI as a predictive tool for AD in its early stages.

The current approaches of AD prediction employ deep learning (DL) and machine learning (ML) algorithms on various imaging and gene expression data. Tanveer et al. (2020) have reviewed machine learning models for neuroimaging analysis, focusing on the prediction of AD. Many such classification studies were dominated with structural data, even though there were studies employing functional data as well. A brief summary of such approaches is described in Table 1. These findings suggest the potential for highly accurate early detection of AD. Arguing that FC networks based on pairwise correlations may rather follow a higher-order relationships, attempts have been made to propose hyperconnectivity network (HCN) models (Guo et al., 2017; Li et al., 2018; Liu et al., 2024a,b). Very recently, many novel methods such as spatio-temporal weighted multi-hypergraph convolutional network (STW-MHGCN), directed hypergraph convolutional network (DHGCN) etc., have been proposed and tested for MCI, AD and Major depressive disorders (MDD) with an impressive success (Liu et al., 2024a,b).

**TABLE 1:** ML and DL-based models and statistics in previous studies.

Research study	Dataset	Models	Average classification accuracy
Abrol et al., 2020	ADNI	CNN +3D ResNet	83%
Saratxaga et al., 2021	OASIS	DL and image processing	88%
Sudharsan and Thailambal, 2023	ADNI	ML	75%
Basheer et al., 2021	OASIS	Deep neural networks	92%
Martinez-Murcia et al., 2020	ADNI	DL using convolutional autoencoders	80%
Liu et al., 2024a	ADNI	Deep spatio-temporal feature fusion (STW-MHGCN)	87%
Prajapati et al., 2021	ADNI	Deep neural network binary classifier	85%
Current study	OASIS	SVM^#^	92%

^#^Only the best model is included in this table.

Our goal is to identify biomarkers in fMRI-derived connectome for an early diagnosis of AD. By applying ML tools to Adult, Aging & AD cohorts, we here provide a method that potentially improves classification, utilizing the graph theory matrices derived from rs-fMRI data. Brain regions categorized in a brain atlas, AAL3 (Rolls et al., 2020) known to be involved in AD pathophysiology were considered and their BOLD time series and correlation matrices were extracted (Lodha et al., 2018). We then applied a threshold to obtain binary FC networks and computed their graph metrics and then used these metrics as features to classify dataset of healthy adults, aging individuals and AD patients.

## 2 Materials and methods

### 2.1 Data compilation

fMRI data for healthy adults, and aging groups were compiled from various sources.

**Data of Adult & Aging individuals:** The fMRI data of adult & aging individuals used in this study, Southwest University Adult Lifespan Dataset (SALD) were collected from 1000 Functional Connectomes Project (FCP) and its successor, the International Neuroimaging Data-Sharing Initiative (INDI). Data details and fMRI acquisition parameters are given in the (Wei et al., 2018). We divided the data into two age groups, one Adult (age 21–50 years), and another, Aging (53–76 years). These age ranges were chosen to capture a broad spectrum of adult development, encompassing both young adult and aging individuals. The Adult group included 40 subjects, while the aging group contained 36 subjects and all of them were healthy subjects. The selection of healthy subjects in the SALD database was based on the following exclusion criteria to avoid medications or co-morbidities: i) MRI-related exclusion criteria, which included claustrophobia, metallic implants, Meniere’s Syndrome and recent (6-months) history of fainting; ii) current psychiatric disorders or neurological disorders; iii) use of psychiatric drugs within the 3 months prior to scanning; iv) pregnancy; or v) a history of head trauma.

**AD data:** The fMRI data of AD individuals, used in this study were collected from the Open Access Series of Imaging Studies (OASIS) dataset (Marcus et al., 2010; LaMontagne et al., 2019). The OASIS is publicly available neuroimaging dataset of healthy adults and individuals with AD. We specifically focused on the data from AD patients within the aging subject, comprising 36 participants, age 64–95 years. Detail of data acquisition in OASIS are available at https://sites.wustl.edu/oasisbrains/. A brief information is provided here: AD diagnosis of subjects based on clinical information, including gradual memory decline and functional impairment MRI scan detail, Siemens scanners, 3T with 16-channel head coil, structural sequences (T1, T2, FLAIR) and functional sequences (resting-state BOLD, ASL). Resting state scans labeled according to BIDS standard “task-rest” (Marcus et al., 2010; LaMontagne et al., 2019).

### 2.2 Image processing

All downloaded data were preprocessed with the CONN-fMRI functional connectivity toolbox (Whitfield-Gabrieli and Nieto-Castanon, 2012) and Statistical Parametric Mapping (SPM) (Eickhoff et al., 2005) with MATLAB R2018b by using the CONN default preprocessing pipeline. All functional images were realigned, unwarped, slice-time corrected, co-registered with structural data, spatially normalized into the standard Montreal Neurological Institute (MNI) space, outlier detected (ART-based scrubbing), and smoothed using a 6mm FWHM Gaussian kernel. Structural data were segmented into gray matter, white matter (WM), and CSF, and normalized in the same default preprocessing pipeline. Region-wise BOLD time-series data from 166 ROIs (Region of Interest) were processed as defined by the Automated Anatomic Labeling atlas (AAL3; Rolls et al., 2020). The AAL3 atlas divides the brain into 166 distinct anatomical regions. These ROIs were further grouped into broader anatomical areas for the present analysis of AD. They are various brain lobes (frontal, parietal, occipital, temporal, cerebellum, and thalamus), important RSN (DMN and FPN) and brain regions such as anterior cingulate cortex. More information on this organization and AAL atlas regions can be found in the Supplementary Table 3. The average BOLD time series for each region was extracted using the AAL3 atlas. The correlation coefficients between each seed-averaged BOLD time series and the BOLD time series of all whole-brain voxels were calculated to create functional connectivity maps from ROI to ROI using the CONN toolbox.

For each ROI, connectivity matrices were created and analyzed using graph theory with the CONN-fMRI toolbox (Whitfield-Gabrieli and Nieto-Castanon, 2012). The ROI-to-ROI study was performed by calculating statistics for all potential links for a subset of ROIs,. In CONN toolbox, thresholding refers to the process of converting a weighted functional connectivity (FC) network into a binary network. This means connections (edges) between brain regions (ROIs) are either considered “present” (connected) or “absent” (not connected) based on a chosen threshold value. We used a threshold of 0.15. This selection aims to balance capturing strong, relevant connections while minimizing weak or spurious ones. Choosing a very high threshold might exclude important connections, while a very low threshold could introduce noise and irrelevant connections. The value of 0.15 is a common choice in the field (Whitfield-Gabrieli and Nieto-Castanon, 2012). The p-FDR (False Discovery Rate) correction was applied to control the false discovery rate when performing multiple comparisons, as described by Benjamini and Hochberg (1995) using CONN toolbox utility. This is a statistical method, which is used to correct for the likelihood of false positives, when conducting multiple hypothesis tests.

### 2.3 Network feature selection

Graph theory-based network parameters have been evaluated for connectomes to study the topological organization of the brain. As mentioned above, the brain was divided into 166 nodes corresponding to the 166 ROIs in AAL3 atlas (Supplementary Table 5). Pertinent to note that the total number of parcellations in AAL3 is 166 having the maximum label number 170. The anterior cingulate cortex (no. 35, 36) and thalamus (no. 81, 82) in previous version of atlas, AAL2 have been left empty in AAL3, since finer parcellations of these regions were provided in AAL3 (Rolls et al., 2020).

For each node, six local graph metrics were calculated, which are average path length (APL), betweenness centrality (BC), clustering coefficient (CC), degree centrality (DC) or cost, global efficiency (GE) and local efficiency (LE) (Achard and Bullmore, 2007). The definition of these parameters, along with formulae are described in the Supplementary Table 4. We obtained a total of 996 features (166 ROIs x 6 network parameters) for each subject. To reduce the dimensionality and select the most relevant features for classification, we used a random forest algorithm for feature selection (Pedregosa et al., 2011). Random forest algorithm is a machine learning technique that uses an ensemble of decision trees to rank the features based on their importance and accuracy.

### 2.4 Machine learning

In this study we employed different machine learning algorithms from the Scikit-learn library to classify the data and identify the optimal model parameters. The algorithms implemented such as Random Forest (Breiman, 2001), Logistic Regression (Wang et al., 2019), XGBoost v1.7.6 (Chen and Guestrin, 2016) and Support Vector Machine (SVM) (Pradhan, 2012), using Pandas v1.5.1, matplotlib v3.5.1, NumPy v1.23.5, SciPy v1.10.1, Scikit-learn v1.1.2, and seaborn v0.12.1 (Pedregosa et al., 2011). In all the models, the datasets were divided into training and test sets, in 80:20 ratio.

To evaluate the best classification model, a variety of algorithms with different hyperparameters were used. For Random Forest, 100 decision trees (n_estimators = 100) was used to improve robustness and combat overfitting. To obtain class probabilities in SVM, probability estimates (probability = True) were enabled. In Logistic Regression, L2 regularization was used to manage model complexity. In XGBoost, we used its default hyperparameter selection to optimize performance. Each algorithm’s performance was evaluated using accuracy, precision, specificity, recall, and F1 score.

### 2.5 Cross validation

To ensure the generalizability and robustness of developed models, a rigorous k-fold cross-validation approach was employed. In this technique, the training data is systematically divided into k equal subsets, or “folds.” The model is then trained k times, each time using a different fold for validation and the remaining folds for training (Berrar, 2019). In our case, we utilized *k* = 5, i.e., the training dataset was split into 5 equal subsets, and the model was trained and evaluated five times, providing a comprehensive assessment of its performance across different data distributions.

All python codes implemented in the present work and associated data are available upon request from authors. Sensitivity analysis were assessed to examine the robustness of the models to variations in feature compositions. The results shown in Supplementary Figure 1 and Supplementary Table 2 highlight the good performance. The circular plots were drawn using CONN toolbox.

## 3 Results and discussion

In the present study, publicly available rs-fMRI datasets, SALD and OASIS, were examined, which cover different stages of cognitive decline of the lifespan dataset. The demographic details are summarized in Table 2. In view of paucity of fMRI data, several studies in the past have utilized data from multiple sources or by concatenating the data (Guo et al., 2017; Liu et al., 2021). Recently, a systematic attempt has been made to evaluate results, derived from concatenated data obtained from multiple sources. The study found that concatenating segments from the same state had a clear advantage over concatenating segments from different states (Cho et al., 2021). The present research is aimed at employing ML and graph theory metrics derived from fMRI data to predict AD and unravel the changes in the functional connectome. We calculated the following metrics for all the generated models, accuracy, recall, specificity, precision, and F1 score (Table 3).

**TABLE 2:** Demographic details of datasets used in the present study^#^.

Data	Subjects	Gender (female, male)	Age range
SALD (adult)	40	22F, 18M	21–50
SALD (aging)	36	22F, 14M	53–76
OASIS (AD)	36	18F,18M	64–95

^#^Further information is given in section “2 Materials and methods.”

**TABLE 3:** Model statistics (5-fold cross validation) as obtained from different ML algorithms^#^.

Model	Accuracy	Sensitivity/recall	Specificity	F1 score	Precision
SVM	92 ± 1.78	78 ± 6.54	99 ± 2.66	86 ± 3.29	97 ± 5.7
Logistic regression	91.94 ± 3.2	83 ± 4.28	96 ± 3.2	87 ± 4.65	91 ± 6.99
Random forest	87.35 ± 3.48	66 ± 11.86	97 ± 3.26	76 ± 7.67	93 ± 8.59
XGBoost	82.85 ± 4.55	58 ± 13.6	95 ± 2.66	68 ± 11.02	84 ± 9.17

^#^Recall, also known as sensitivity, refers to the percentage of individuals correctly identified as having Alzheimer’s disease. Precision pertains to the accuracy with which the diagnosis excludes individuals without the disease. The F1 score is a balanced mean of precision and recall, whereas accuracy indicates the overall rate of correct classification among the population.

**FIGURE 1:**
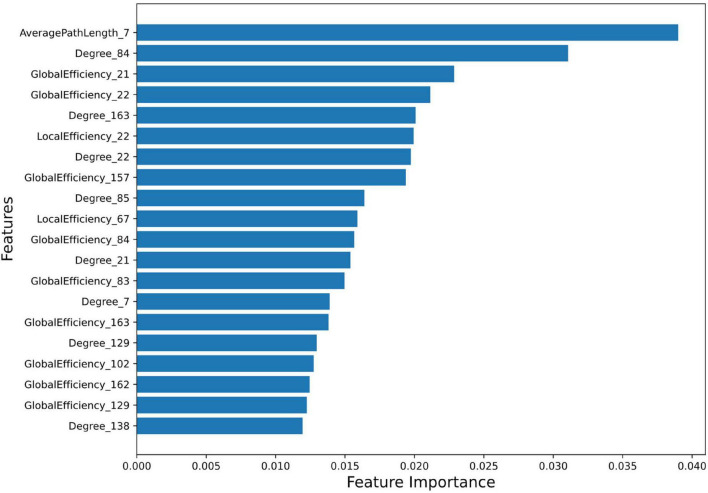
Feature Importance plot using Random Forest method. The plot shows the relative importance of each feature on the horizontal axis, and the names of the features on the vertical axis. The detail description of AAL3 region numbers are provided in Supplementary Table 5.

*Feature Importance and Ranking:* Feature importance score of all the 996 features were calculated using random forest algorithm. A total of 265 features were selected using the Random Forest feature selection method. Feature importance signifies the contribution of each feature to predict AD using the random forest regressor (score values of top 20 features (top20) and all 265 features are provided in Supplementary Tables 6, 7, respectively). Feature importance values indicate their greater significance in the predictive model and they play essential role in the model’s decision-making. Feature importance plot (Figure 1) visualizes the significance of top20 features in predicting outcomes.

A brief summary of all ML models using 5-fold cross-validation is described in Table 3 (and Supplementary Tables 1, 2). All the models demonstrated high accuracy, sensitivity, precision and specificity, suggesting their potential for accurate AD prediction. All the models achieved reasonably good performance, with SVM attaining the highest accuracy of 92%, followed by Logistic Regression. The Random Forest and XGBoost model had a slightly lower accuracy of 87.4% and 82.9%, respectively. The high performance scores with SVM could be due to its ability to deal with complex, high-dimensional datasets, and avoid over-fitting (Noble, 2006; Han and Jiang, 2014).

The top20 features corresponding to the most important 13 regions (top13ROI) are as follows: left inferior frontal gyrus, opercular part (abbreviated as IFG-L, AAL3 atlas region, Frontal_Inf_Oper_L 7), bilateral Heschl’s gyri (HG-R and HG-L, Heschl_R 84/L83), bilateral superior frontal gyri, medial orbital (SFG-L and SFG-R, Frontal_Med_Orb_L 21/R_22), left substantia nigra, pars reticulata (SNr-L, SN_pr_L163), left nucleus accumbens (NAc-L, N_Acc_L157), left superior temporal gyrus (STG-L, Temporal_Sup_L 85), left supramarginal gyrus (SMG-L, SupraMarginal_L 67), left ventral posterolateral of thalamus (VPL-L, Thal_VPL_L 129), right cerebellar hemisphere (lobule IV-V) (CER-R IV-V, Cerebellum_4_5_R 102), right substantia nigra, pars compacta (SNc-R, SN_pc_R162) and right mediodorsal lateral parvocellular of thalamus (MDl-R, Thal_MDl_R 138).

**FIGURE 2:**
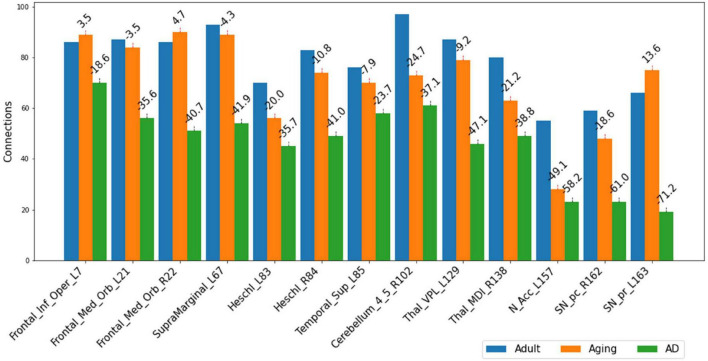
Number of connections among three different cohorts, Adult, Aging and AD, between each of the important 13-regions (top13ROI) and rest of the ROI in AAL3 atlas. Numerals on top of each bar indicate percent of connection changes with respect to Adult (considered as 100%). Negative values correspond to drop in connections & vice versa for positive values. On the *x*-axis, AAL3 atlas labels are indicated in order and their corresponding regions are described in text (section “3 Results and discussion”).

The present study attempts to decipher the changes occurring in functional connectome as a result of AD using ML approaches and graph theory. To understand the mechanistic point of view of these important regions in AD as revealed from the present study, we therefore examined their connectivity patterns. Upon observation of the total number of connections within top13ROI, significant reductions were noticed in AD data as compared to Adult and Aging for all these 13 regions. The drop in these regions in AD (as compared to Adult) were observed as much as 70%. Except IFG-L and STG-L, substantial drop (∼35–70%) were observed in almost all the regions. As much as 60–70% reductions were noticed in SNr-L, SNc-R and NAc-L and about 50% for VPL-L (Figure 2). In contrast, in Aging data (as compared to Adult), maximum 50% reduction was found for NAc-L and even slight gain were observed for few regions. The massive disruptions of functional connections occurring in these regions of the brain among AD cohorts corroborate with the previous studies which is suggestive of a crucial biomarker of the disease. SNc-R and SNr-L located in the midbrain, plays a crucial role in dopamine production, which is essential for movement control and coordination (Sonne et al., 2023). Both the regions are also involved in the mesostriatal and mesolimbocortical systems, which are related to sensorimotor processing and limbic mechanisms. A previous study focusing on AD and Parkinson’s disease indicated that the number of neurons were reduced by 78–97% as compared to control in the medial to lateral substantia nigra, pars compacta (Zarow et al., 2003) Furthermore, it has been found that AD patients showed significant reductions in the left and right nucleus accumbens volumes (Pievani et al., 2013). Another study have demonstrated significantly reduced cortical thickness and surface area in these regions (Yang et al., 2019). Similarly, VPL and MDl of thalamus act as a relay station, sending sensory information from the body to the cortex. Medidorsal thalamus has been previously implicated in modulation of cognitive performance (Ferguson and Gao, 2015). In AD, the VPL atrophy was also observed in previous studies (Paskavitz et al., 1995; Forno et al., 2023). Heschl’s gyrus possess strong and positive functional connectivity with many regions involved in sensory, sensorimotor, and cognitive brain networks. Altered functional connectivity has been observed in right Heschl’s gyrus (Fitzhugh et al., 2019; Biswas and Sripada, 2023). The IFG, which is a part of Broca’s region is essential for language generation and voice processing. It also helps to understand voice tones in spoken languages. Research on, AD patients had found reduced gray matter volume and altered functional connections in the right opercular portion (Thompson et al., 2003; Vidoni et al., 2010; Zhu et al., 2023).

**FIGURE 3:**
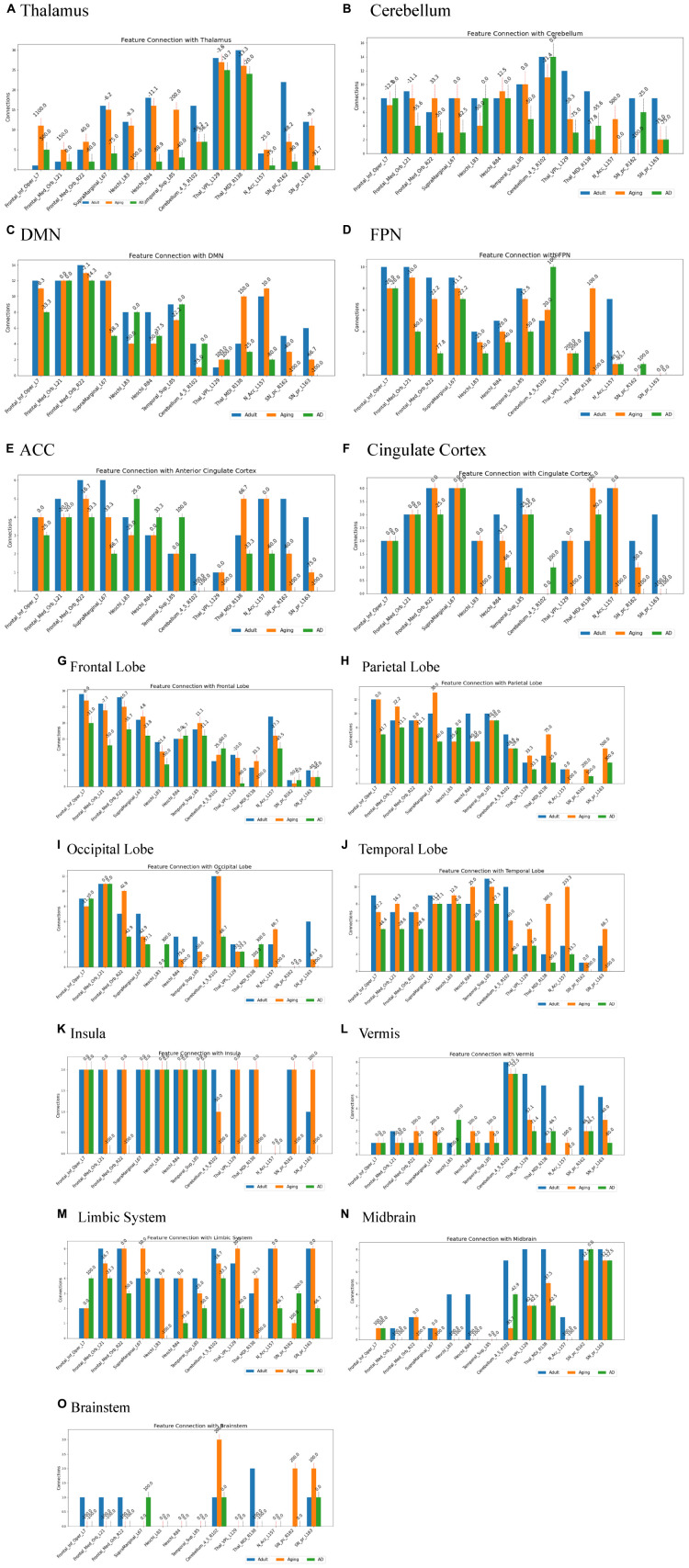
Number of connections among three different cohorts, Adult, Aging and AD, between group-1 (top13ROI) and group-2 [**(A)** thalamus, **(B)** Cerebellum, **(C)** DMN, **(D)** FPN, **(E)** ACC, **(F)** Cingulate Cortex, **(G)** Frontal Lobe, **(H)** Parietal Lobe, **(I)** Occipital Lobe, **(J)** Temporal Lobe, **(K)** Insula, **(L)** Vermis, **(M)** Limbic System, **(N)** Midbrain, and **(O)** Brainstem implicated in AD], Numerals on top of each bar indicate percent of connection changes with respect to adult (considered as 100%). On the x-axis, AAL3 atlas labels are indicated in order and their corresponding regions are described in text (section “3 Results and discussion”).

**FIGURE 4:**
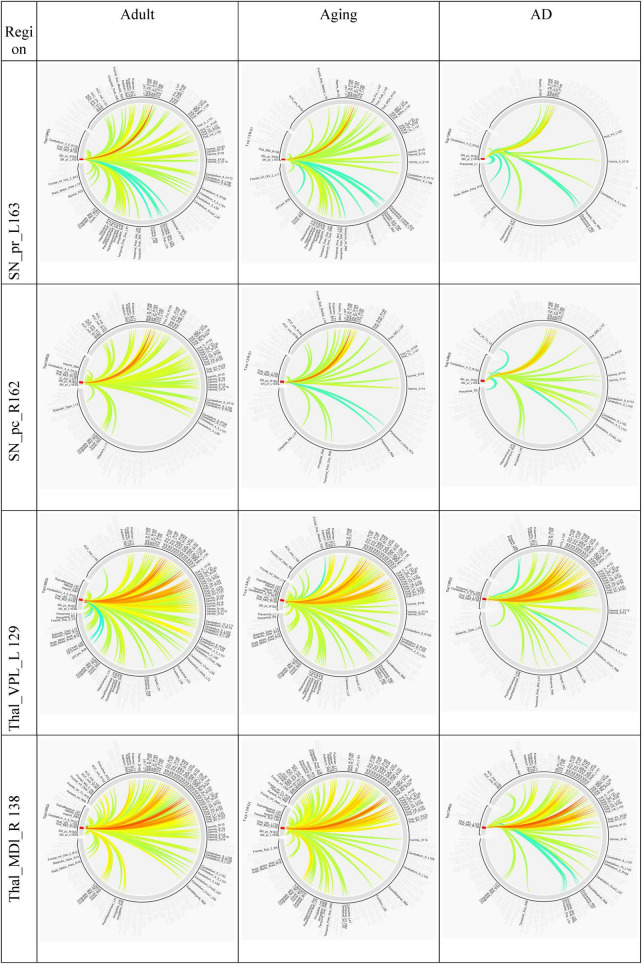
Circular plot of connections among three different cohorts, Adult, Aging and AD, for SNr-L, SNc-R, VPL-L and Mdl-R. AAL3 atlas labels are indicated on the circumference and their corresponding regions are described in text (section “3 Results and discussion”).

**FIGURE 5:**
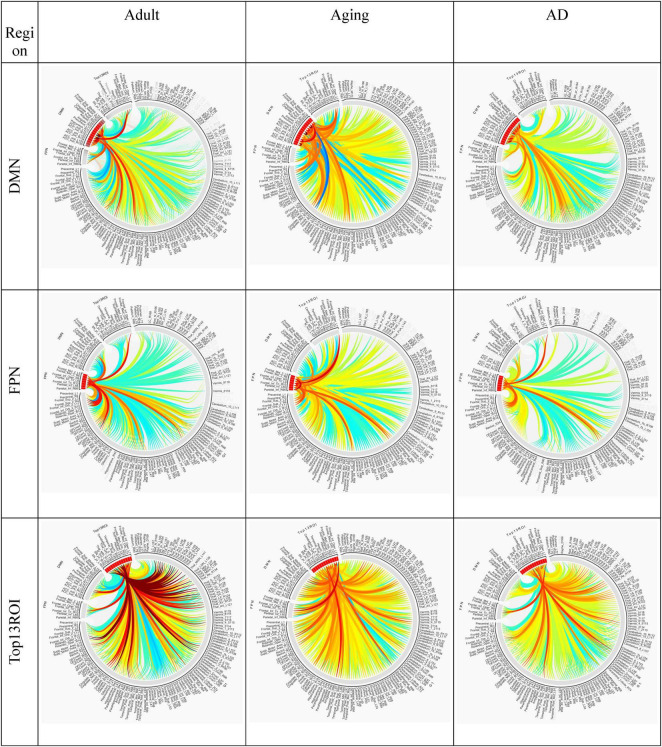
Circular plot of connections among three different cohorts, Adult, Aging and AD, for two important resting-state cognitive networks, DMN, and FPN and top13ROI. AAL3 atlas labels are indicated on the circumference and their corresponding regions are described in text (section “3 Results and discussion”).

Several studies on AD in the past have focussed on examination of role of RSNs, particularly DMN and FPN in cognition, and their altered FC (Badhwar et al., 2017; Contreras et al., 2019; Farràs-Permanyer et al., 2019). Hence, it would be worthwhile to examine changes in the connection patterns between top13ROI and individual RSNs (DMN and FPN), as well as with different brain lobes (frontal, parietal, occipital, temporal, cerebellum, and thalamus) and important regions, which were previously implicated in AD. Supplementary Table 3 lists these groups and included AAL3 atlas regions. These connections were computed between top13ROI and all the regions defined in Supplementary Table 3. Figure 3 highlights relative strengths of functional connections among Adult, Aging and AD data. The trend is similar to general connectivity pattern of top13ROI as described in Figure 2. In all the bar graphs Figures 3A–O, significant drop in connections for SNr-L, SNc-R and NAc-L have been observed. As shown in Circular plot (Figures 4, 5) and bar graph (Figure 3A), majority of losses for top13ROI regions (prominently for SNr-L, SNc-R, NAc-L and HG-L/R and SMG-L) were noticed with the thalamus region. It is striking to note that connection strengths with top13ROI has dramatically gone up (Figure 5) in Aging data in comparison to Adult, while decline was seen in AD data. It appears that such hyperactivation in the certain cortical and sub-cortical regions among healthy aging individuals may act as a compensatory mechanism to cope with the challenges faced by declining brain (Dickerson and Sperling, 2008). Aging individuals lacking this lead to such neurodegenrative disorders.

Two of the RSN, DMN and FPN have received enormous attention in biomarker development for AD (Badhwar et al., 2017; Farràs-Permanyer et al., 2019; Zhukovsky et al., 2023). AD patients exhibit a broad decline in brain activity consistent with symptoms such as memory loss, with the largest reductions occurring in regions associated with the DMN. Studies indicate that AD pathology could initiate in the DMN prior to the onset of clinical symptoms, hence giving rise to the hypothesis that malfunctions in this network could play a pivotal role in the advancement of the illness. Similarly significantly loss of FC in FPN, which is strongly associated with executive function and cognitive control were reported (Marek and Dosenbach, 2018; Wei et al., 2018). In the present work, substantial loss of top13ROI with DMN were observed primarily for SNr-L, SNc-L, NAc-L and MDl-R, and with FPN losses were noticed for MDl-R, NAc-L and SFG-L/R (Figure 3).

It has also been observed that early symptoms of AD appear as atrophy in the ACC, which is also an important component of the DMN (Xu et al., 2016; Chen et al., 2021; Tu et al., 2021). This atrophy has been frequently observed as an early symptom in clinical investigations (Lee et al., 2020; Yuan et al., 2022). Hence, we specifically examined ACC and rest of the cingulate cortex (PCC and MCC) with top13ROI. Our findings show a considerable decline in network connectivity, particularly in SNr-L, SNc-R, NAc-L and VPL-L (Figure 3). Furthermore, decline in connections were also noticed for Insula, Cerebellum & Vermis for SNr-L, SNc-R, VPL-L and MDl.

Based on these patterns, it appears that both cortical and subcortical parts of neural networks are widely disrupted in AD, which may explain some of the disease’s complicated symptoms. The present study, highlighting that connection decline with substantia nigra, pars reticulata, substantia nigra, pars compacta, and nucleus accumbens could be a potential biomarker for early prediction of AD.

## 4 Conclusion and limitation of the study

In the present study, using graph theory metrics derived from rs-fMRI data and machine learning, attempt has been made to identify key features in the functional connectome that could serve as biomarkers to predict AD. Utilizing 5-fold cross-validation, the ML models demonstrated high accuracy, sensitivity, specificity, and precision, and the SVM model demonstrated the highest accuracy of 92%, proving its robustness in generalizing new data without overfitting. The study highlights that left inferior frontal gyrus, opercular part, bilateral Heschl’s gyri, bilateral superior frontal gyri, medial orbital, left substantia nigra, pars reticulata, left nucleus accumbens, left superior temporal gyrus, left supramarginal gyrus, left ventral posterolateral of thalamus, right cerebellar hemisphere (lobule IV-V), right substantia nigra, pars compacta and right mediodorsal lateral parvocellular of thalamus are the most important regions for AD. In these regions, connection strengths with other regions of connectome has substantially dropped. In particular drastic reductions were noticed for substantia nigra, pars reticulata, substantia nigra, pars compacta, nucleus accumbens and ventral posterolateral of thalamus among AD patients. Further, prominent and consistent loss of functional connections between these 13 regions and the thalamus is another noteworthy indication of this study. The present findings corroborate with the earlier studies, employing various neuroimagining techniques. The present investigation is a comprehensive approach, integrating ML, graph theory, and rs-fMRI data analysis to identify distinct regions in AD subjects in comparision to healthy adults and Aging individuals. The significant loss in these regions could be a potential biomarker, which may improve early diagnosis and intervention strategies for AD.

Despite that, the present study is limited in may aspects. The study depends on a limited number of publicly available fMRI datasets, which may introduce bias, as these datasets might not fully represent the population’s diversity, and consequently, may affect the generalizability of the results. The study also uses a small independent validation dataset, which lowers the statistical power and the model applicability. Additionally, it has also been argued that pairwise correlations based functional connectivity networks ignores higher-order relationships, and may not effectively characterize the high-order interacons of many brain regions. However, hypergraph modeling networks are very noise sensitive limiting its applications (Dai and Gao, 2023). Recently several attempts have been made toward this direction (Liu et al., 2024a,b). Concern has also been raised with the choice of atlas on result variability as different brain atlases lead to different partitions. However, earlier attempt to carry out similar study on MCI using different atlases noticed not much differences lying within few percent (Long et al., 2018). More research is needed to examine how the brain network characteristics are associated with the disease progression and symptoms. In conclusion, our study uncovers the important regions using machine learning and graph theory, which certainly has the potential to server biomarkers for prediction of AD.

## Data availability statement

The original contributions presented in this study are included in this article/Supplementary material, further inquiries can be directed to the corresponding author.

## Ethics statement

Written informed consent was obtained from the minor(s)’ legal guardian/next of kin for the publication of any potentially identifiable images or data included in this article.

## Author contributions

SK: Writing – review and editing, Writing – original draft, Visualization, Validation, Software, Resources, Methodology, Investigation, Formal analysis, Data curation, Conceptualization. RR: Writing – review and editing, Investigation, Formal analysis Validation, Supervision, MF: Methodology, Investigation, Formal analysis.
