# Supplementary material for: Identifying discriminative features of brain network for prediction of Alzheimer’s disease using graph theory and machine learning
Source: Front Neuroinform. 2024 Jun 18;18:1384720. doi: 10.3389/fninf.2024.1384720 (PMC11217540; doi:10.3389/fninf.2024.1384720)
Supplement: Supplementary file 1 [file Data_Sheet_1.docx]

**Identifying discriminative features of brain network for prediction of Alzheimer's disease using graph theory and machine learning**

S. M. Shayez Karim^1^, Md Shah Fahad^2,^ and R. S. Rathore^1*^

^1^ Department of Bioinformatics, Central University of South Bihar, Gaya, Bihar 824236, India.

^2^ Department of Computer Science And Engineering, Birla Institute of Technology, Mesra, Ranchi, India

*Corresponding author: rsrathore@cusb.ac.in

**Supplementary Material**

*Table 1: Training vs. Validation performance of Machine Learning models*

| ‍‍Score | SVM | Logistic Regression | Random Forest | XGBoost |
| --- | --- | --- | --- | --- |
| Training Accuracy | 97.75 | 100 | 100 | 100 |
| Validation Accuracy | 91.9 | 91 | 87.35 | 82.85 |
| Training Precision | 100 | 100 | 100 | 100 |
| Validation Precision | 97 | 91 | 93 | 84 |
| Training Specificity | 100 | 100 | 100 | 100 |
| Validation Specificity | 99 | 96 | 97 | 95 |
| Training F1 Score | 96 | 100 | 100 | 100 |
| Validation F1 Score | 86 | 87 | 76 | 68 |
| Training Recall | 96 | 100 | 100 | 100 |
| Validation Recall | 78 | 83 | 66 | 58 |

*Table 2: Feature sensitivity performance of Machine Learning models (F – feature)*

| **Model** | **Accuracy** | | | |
| --- | --- | --- | --- | --- |
|  | F 0: 70 | F 70:140 | F 140:210 | F 210:265 |
| **SVM** | 91.3 | 87 | 78.2 | 74 |
| Logistic Regression | 82 | 74 | 82 | 69 |
| Random Forest | 87 | 74 | 78 | 78 |
| XGBoost | 82 | 69 | 82 | 82 |

*Figure 1: Feature sensitivity performance of Machine Learning models with different sets of features (F – feature)*

*Table 3: List of 166 anatomical regions of the brain in AAL3 atlas. These atlas regions have been grouped into anatomical areas (lobes) - frontal, parietal, occipital, temporal, cerebellum, thalamus, Cingulate Cortex, Anterior Cingulate Cortex, Default Mode Network (DMN), Fronto-parietal Network (FPN) as per the lobe-wise region assignment made in PMOD software (Oliver et al., 2019; PMOD Technologies LLC* – PMOD Technologies, n.d.)*.*

| **Frontal Lobe**  Precentral_L1  Precentral_R2  Frontal_Sup_2_L3  Frontal_Sup_2_R4  Frontal_Mid_2_L5  Frontal_Mid_2_R6  Frontal_Inf_Oper_L7  Frontal_Inf_Oper_R8  Frontal_Inf_Tri_L9  Frontal_Inf_Tri_R10  Frontal_Inf_Orb_2_L11  Frontal_Inf_Orb_2_R12  Rolandic_Oper_L13  Rolandic_Oper_R14  Supp_Motor_Area_L15  Supp_Motor_Area_R16  Olfactory_L17  Olfactory_R18  Frontal_Sup_Medial_L19  Frontal_Sup_Medial_R20  Frontal_Med_Orb_L21  Frontal_Med_Orb_R22  Rectus_L23  Rectus_R24  OFCmed_L25  OFCmed_R26  OFCant_L27  OFCant_R28  OFCpost_L29  OFCpost_R30  OFClat_L31  OFClat_R32  **Parietal Lobe**  Postcentral_L61  Postcentral_R62  Parietal_Sup_L63  Parietal_Sup_R64  Parietal_Inf_L65  Parietal_Inf_R66  SupraMarginal_L67  SupraMarginal_R68  Angular_L69  Angular_R70  Precuneus_L71  Precuneus_R72  Paracentral_Lobule_L73  Paracentral_Lobule_R74 | **Occipital Lobe**  Calcarine_L47  Calcarine_R48  Cuneus_L49  Cuneus_R50  Lingual_L51  Lingual_R52  Occipital_Sup_L53  Occipital_Sup_R54  Occipital_Mid_L55  Occipital_Mid_R56  Occipital_Inf_L57  Occipital_Inf_R58  **Temporal lobe**  Fusiform_L59  Fusiform_R60  Heschl_L83  Heschl_R84  Temporal_Sup_L85  Temporal_Sup_R86  Temporal_Pole_Sup_L87  Temporal_Pole_Sup_R88  Temporal_Mid_L89  Temporal_Mid_R90  Temporal_Pole_Mid_L91  Temporal_Pole_Mid_R92  Temporal_Inf_L93  Temporal_Inf_R94  **Cerebellum**  Cerebellum_Crus1_L95  Cerebellum_Crus1_R96  Cerebellum_Crus2_L97  Cerebellum_Crus2_R98  Cerebellum_3_L99  Cerebellum_3_R100  Cerebellum_4_5_L101  Cerebellum_4_5_R102  Cerebellum_6_L103  Cerebellum_6_R104  Cerebellum_7b_L105  Cerebellum_7b_R106  Cerebellum_8_L107  Cerebellum_8_R108  Cerebellum_9_L109  Cerebellum_9_R110  Cerebellum_10_L111  Cerebellum_10_R112  **CingulateCortex**  Cingulate_Mid_L37  Cingulate_Mid_R38  Cingulate_Post_L39  Cingulate_Post_R40  Limbic System  Hippocampus_L41  Hippocampus_R42  ParaHippocampal_L43  ParaHippocampal_R44  Amygdala_L45  Amygdala_R46 | **FPN**  Frontal_Mid_2_L5  Frontal_Mid_2_R6  Frontal_Inf_Oper_L7  Frontal_Inf_Oper_R8  Frontal_Inf_Tri_L9  Frontal_Inf_Tri_R10  Parietal_Inf_L65  Parietal_Inf_R66  Angular_L69  Angular_R70  **DMN**  Frontal_Sup_Medial_L19  Frontal_Sup_Medial_R20  ACC_sub_L151  ACC_sub_R152  ACC_pre_L153  ACC_pre_R154  ACC_sup_L155  ACC_sup_R156  Cingulate_Post_L39  Cingulate_Post_R40  Angular_L69  Angular_R70  Precuneus_L71  Precuneus_R72  **Brainstem**  LC_L167  LC_L168  Raphe_D169  Raphe_M170  **Vermis**  Vermis_1_2113  Vermis_3114  Vermis_4_5115  Vermis_6116  Vermis_7117  Vermis_8118  Vermis_9119  Vermis_10120  **Limbic System**  Hippocampus_L41  Hippocampus_R42  ParaHippocampal_L43  ParaHippocampal_R44  Amygdala_L45  Amygdala_R46  **Insula**  Insula_L33  Insula_R34 | **Thalamus**  Thal_AV_L121  Thal_AV_R122  Thal_LP_L123  Thal_LP_R124  Thal_VA_L125  Thal_VA_R126  Thal_VL_L127  Thal_VL_R128  Thal_VPL_L129  Thal_VPL_R130  Thal_IL_L131  Thal_IL_R132  Thal_Re_L133  Thal_Re_R134  Thal_MDm_L135  Thal_MDm_R136  Thal_MDl_L137  Thal_MDl_R138  Thal_LGN_L139  Thal_LGN_R140  Thal_MGN_L141  Thal_MGN_R142  Thal_PuA_L143  Thal_PuA_R144  Thal_PuM_L145  Thal_PuM_R146  Thal_PuL_L147  Thal_PuL_R148  Thal_PuI_L149  Thal_PuI_R150  **Midbrain**  VTA_L159  VTA_R160  SN_pc_L161  SN_pc_R162  SN_pr_L163  SN_pr_R164  Red_N_L165  Red_N_R166  **Anterior Cingulate Cortex**  ACC_sub_L151  ACC_sub_R152  ACC_pre_L153  ACC_pre_R154  ACC_sup_L155  ACC_sup_R156 |
| --- | --- | --- | --- |

*Table 4: Description of the graph theory parameters used in our study*

| Parameters | Description |
| --- | --- |
| Average Path Length | The minimum number of edges that must be traversed to go from one node to another. It is used as a measure of global integration of the network  $L_{i}=\frac{\sum_{j\in\Omega_{i}} D_{i,j}}{N_{i}-1}$  $L=\frac{\sum_{i} L_{i}}{N}$  where D is the shortest-path distance matrix, N is the total number of nodes in a graph, and L is the averages path distance of a graph (and of each individual node/ROI). The index i refers to the individual node, which varies from 1 to N. |
| Betweenness Centrality | Used to investigate the contribution of each node to all other node pairs on the shortest path. It measures not only the importance of the nodes, but also the amount of information flowing through the node  $BC_{i}=\frac{\sum_{j,k\neq i} \left[ i\in P_{j,k} \right]}{\left( N-1 \right)\left( N-2 \right)}$  $BC=\frac{\sum_{i} BC_{i}}{N}$  where P is the set of nodes in shortest-path between each pair of nodes, N is the number of nodes in a graph, and BC is the Betweenness Centrality of a graph (and of each individual node/ROI) |
| Clustering Coefficient | The number of connections that exist between the nearest neighbors of a node as a proportion of maximum number of possible connections. It reflects the tendency of a network to form topologically organized circuits and it is often interpreted as a metric of information segregation in networks  $CC_{i}=\frac{\sum_{j,k\in\Gamma_{i}} A_{j,k}^{\left( i \right)}}{d_{i}\left( d_{i}-1 \right)}$  $CC=\frac{\sum_{i} CC_{i}}{N}$  where d is the degree of each node, A is the adjacency matrix within the neighboring sub-graph at each node, characterized by all nodes neighboring this node and all existing edges among them, and CC is the clustering coefficient of a graph (and of each individual node/ROI) |
| Degree Centrality | The degree of a node is the sum of its incoming (afferent) and outgoing (efferent) edges  $d_{i}=\sum_{j} A_{i,j}$  $d=\frac{\sum_{i} d_{i}}{N}$  where A is an adjacency matrix, N is the total number of nodes in a graph, and d is the degree of a graph (and of each individual node/ROI) |
| Global efficiency | Measure of network integration and its overall performance for information transferring. This measure is inversely related to the average shortest path length  $GE_{i}=\frac{{\sum_{j\neq i} 1}/{D_{i,j}}}{N-1}$ $\frac{\sum_{i} GE_{i}}{N}$  Where D is the shortest-path distance matrix, N is the number of nodes in a graph, and GE is the Global Efficiency of a graph (and of each individual node/ROI). Global efficiency at a node represents a measure of this node centrality within the network, characterizing the degree of global connectedness of each ROI. Similarly, network global efficiency represents a measure of inter-connectedness or radius of the entire network (e.g. with higher / more compact global efficiency in random graphs compared to grids) |
| Local efficiency | Local efficiency, which has a similar interpretation as clustering coefficient, measures the compactness of the subnetwork  $LE_{i}=\frac{{\sum_{j\neq k\in\Gamma_{i}} 1}/{D_{j,k}^{\left( i \right)}}}{d_{i}\left( d_{i}-1 \right)}$ $\frac{\sum_{i} LE_{i}}{N}$  Where d is the degree of each node, D is the shortest-path distance matrix within the neighboring sub-graph at each node, characterized by all nodes neighboring this node and all existing edges among them, and LE is the Local Efficiency of a graph (and of each individual node/ROI). Local efficiency represents a measure of local integration or coherence, characterizing the degree of inter-connectedness among all nodes within a node neighboring sub-graph. Similarly, network local efficiency represents a measure of local integration in a network (e.g. with higher local efficiency in grids compared to random graphs) |

*Table 5: Description with labels of the brain regions (nodes) used in our study*

| AAL 3 Atlas Regions | | |
| --- | --- | --- |
| aal3.1 Precentral_L 1 | aal3.59 Fusiform_L 59 | aal3.117 Vermis_7 117 |
| aal3.2 Precentral_R 2 | aal3.60 Fusiform_R 60 | aal3.118 Vermis_8 118 |
| aal3.3 Frontal_Sup_2_L 3 | aal3.61 Postcentral_L 61 | aal3.119 Vermis_9 119 |
| aal3.4 Frontal_Sup_2_R 4 | aal3.62 Postcentral_R 62 | aal3.120 Vermis_10 120 |
| aal3.5 Frontal_Mid_2_L 5 | aal3.63 Parietal_Sup_L 63 | aal3.121 Thal_AV_L 121 |
| aal3.6 Frontal_Mid_2_R 6 | aal3.64 Parietal_Sup_R 64 | aal3.122 Thal_AV_R 122 |
| aal3.7 Frontal_Inf_Oper_L 7 | aal3.65 Parietal_Inf_L 65 | aal3.123 Thal_LP_L 123 |
| aal3.8 Frontal_Inf_Oper_R 8 | aal3.66 Parietal_Inf_R 66 | aal3.124 Thal_LP_R 124 |
| aal3.9 Frontal_Inf_Tri_L 9 | aal3.67 SupraMarginal_L 67 | aal3.125 Thal_VA_L 125 |
| aal3.10 Frontal_Inf_Tri_R 10 | aal3.68 SupraMarginal_R 68 | aal3.126 Thal_VA_R 126 |
| aal3.11 Frontal_Inf_Orb_2_L 11 | aal3.69 Angular_L 69 | aal3.127 Thal_VL_L 127 |
| aal3.12 Frontal_Inf_Orb_2_R 12 | aal3.70 Angular_R 70 | aal3.128 Thal_VL_R 128 |
| aal3.13 Rolandic_Oper_L 13 | aal3.71 Precuneus_L 71 | aal3.129 Thal_VPL_L 129 |
| aal3.14 Rolandic_Oper_R 14 | aal3.72 Precuneus_R 72 | aal3.130 Thal_VPL_R 130 |
| aal3.15 Supp_Motor_Area_L 15 | aal3.73 Paracentral_Lobule_L 73 | aal3.131 Thal_IL_L 131 |
| aal3.16 Supp_Motor_Area_R 16 | aal3.74 Paracentral_Lobule_R 74 | aal3.132 Thal_IL_R 132 |
| aal3.17 Olfactory_L 17 | aal3.75 Caudate_L 75 | aal3.133 Thal_Re_L 133 |
| aal3.18 Olfactory_R 18 | aal3.76 Caudate_R 76 | aal3.134 Thal_Re_R 134 |
| aal3.19 Frontal_Sup_Medial_L 19 | aal3.77 Putamen_L 77 | aal3.135 Thal_MDm_L 135 |
| aal3.20 Frontal_Sup_Medial_R 20 | aal3.78 Putamen_R 78 | aal3.136 Thal_MDm_R 136 |
| aal3.21 Frontal_Med_Orb_L 21 | aal3.79 Pallidum_L 79 | aal3.137 Thal_MDl_L 137 |
| aal3.22 Frontal_Med_Orb_R 22 | aal3.80 Pallidum_R 80 | aal3.138 Thal_MDl_R 138 |
| aal3.23 Rectus_L 23 | aal3.83 Heschl_L 83 | aal3.139 Thal_LGN_L 139 |
| aal3.24 Rectus_R 24 | aal3.84 Heschl_R 84 | aal3.140 Thal_LGN_R 140 |
| aal3.25 OFCmed_L 25 | aal3.85 Temporal_Sup_L 85 | aal3.141 Thal_MGN_L 141 |
| aal3.26 OFCmed_R 26 | aal3.86 Temporal_Sup_R 86 | aal3.142 Thal_MGN_R 142 |
| aal3.27 OFCant_L 27 | aal3.87 Temporal_Pole_Sup_L 87 | aal3.143 Thal_PuA_L 143 |
| aal3.28 OFCant_R 28 | aal3.88 Temporal_Pole_Sup_R 88 | aal3.144 Thal_PuA_R 144 |
| aal3.29 OFCpost_L 29 | aal3.89 Temporal_Mid_L 89 | aal3.145 Thal_PuM_L 145 |
| aal3.30 OFCpost_R 30 | aal3.90 Temporal_Mid_R 90 | aal3.146 Thal_PuM_R 146 |
| aal3.31 OFClat_L 31 | aal3.91 Temporal_Pole_Mid_L 91 | aal3.147 Thal_PuL_L 147 |
| aal3.32 OFClat_R 32 | aal3.92 Temporal_Pole_Mid_R 92 | aal3.148 Thal_PuL_R 148 |
| aal3.33 Insula_L 33 | aal3.93 Temporal_Inf_L 93 | aal3.149 Thal_PuI_L 149 |
| aal3.34 Insula_R 34 | aal3.94 Temporal_Inf_R 94 | aal3.150 Thal_PuI_R 150 |
| aal3.37 Cingulate_Mid_L 37 | aal3.95 Cerebellum_Crus1_L 95 | aal3.151ACC_sub_L151 |
| aal3.38 Cingulate_Mid_R 38 | aal3.96 Cerebellum_Crus1_R 96 | aal3.152ACC_sub_R152 |
| aal3.39 Cingulate_Post_L 39 | aal3.97 Cerebellum_Crus2_L 97 | aal3.153ACC_pre_L153 |
| aal3.40 Cingulate_Post_R 40 | aal3.98 Cerebellum_Crus2_R 98 | aal3.154ACC_pre_R154 |
| aal3.41 Hippocampus_L 41 | aal3.99 Cerebellum_3_L 99 | aal3.155ACC_sup_L155 |
| aal3.42 Hippocampus_R 42 | aal3.100 Cerebellum_3_R 100 | aal3.156ACC_sup_R156 |
| aal3.43 ParaHippocampal_L 43 | aal3.101 Cerebellum_4_5_L 101 | aal3.157N_Acc_L157 |
| aal3.44 ParaHippocampal_R 44 | aal3.102 Cerebellum_4_5_R 102 | aal3.158N_Acc_R158 |
| aal3.45 Amygdala_L 45 | aal3.103 Cerebellum_6_L 103 | aal3.159VTA_L159 |
| aal3.46 Amygdala_R 46 | aal3.104 Cerebellum_6_R 104 | aal3.160VTA_R160 |
| aal3.47 Calcarine_L 47 | aal3.105 Cerebellum_7b_L 105 | aal3.161SN_pc_L161 |
| aal3.48 Calcarine_R 48 | aal3.106 Cerebellum_7b_R 106 | aal3.162SN_pc_R162 |
| aal3.49 Cuneus_L 49 | aal3.107 Cerebellum_8_L 107 | aal3.163SN_pr_L163 |
| aal3.50 Cuneus_R 50 | aal3.108 Cerebellum_8_R 108 | aal3.164SN_pr_R164 |
| aal3.51 Lingual_L 51 | aal3.109 Cerebellum_9_L 109 | aal3.165Red_N_L165 |
| aal3.52 Lingual_R 52 | aal3.110 Cerebellum_9_R 110 | aal3.166Red_N_R166 |
| aal3.53 Occipital_Sup_L 53 | aal3.111 Cerebellum_10_L 111 | aal3.167LC_L167 |
| aal3.54 Occipital_Sup_R 54 | aal3.112 Cerebellum_10_R 112 | aal3.168LC_R168 |
| aal3.55 Occipital_Mid_L 55 | aal3.113 Vermis_1_2 113 | aal3.169Raphe_D169 |
| aal3.56 Occipital_Mid_R 56 | aal3.114 Vermis_3 114 | aal3.170Raphe_M170 |
| aal3.57 Occipital_Inf_L 57 | aal3.115 Vermis_4_5 115 |  |
| aal3.58 Occipital_Inf_R 58 | aal3.116 Vermis_6 116 |  |

*Table 6: Feature importance of top20 features*

|  | AAL3 Region | Feature importance |
| --- | --- | --- |
| AveragePathLength_7 | Frontal_Inf_Oper_L 7 | 0.04 |
| Degree_84 | Heschl_R 84 | 0.03 |
| GlobalEfficiency_21 | Frontal_Med_Orb_L 21 | 0.02 |
| GlobalEfficiency_22 | Frontal_Med_Orb_R 22 | 0.02 |
| Degree_163 | SN_pr_L163 | 0.02 |
| LocalEfficiency_22 | Frontal_Med_Orb_R 22 | 0.02 |
| Degree_22 | Frontal_Med_Orb_R 22 | 0.02 |
| GlobalEfficiency_157 | N_Acc_L157 | 0.02 |
| Degree_85 | Temporal_Sup_L 85 | 0.02 |
| LocalEfficiency_67 | SupraMarginal_L 67 | 0.02 |
| GlobalEfficiency_84 | Heschl_R 84 | 0.02 |
| Degree_21 | Frontal_Med_Orb_L 21 | 0.02 |
| GlobalEfficiency_83 | Heschl_L 83 | 0.01 |
| Degree_7 | Frontal_Inf_Oper_L 7 | 0.01 |
| GlobalEfficiency_163 | SN_pr_L163 | 0.01 |
| Degree_129 | Thal_VPL_L 129 | 0.01 |
| GlobalEfficiency_102 | Cerebellum_4_5_R 102 | 0.01 |
| GlobalEfficiency_162 | SN_pc_R162 | 0.01 |
| GlobalEfficiency_129 | Thal_VPL_L 129 | 0.01 |
| Degree_138 | Thal_MDl_R 138 | 0.01 |

*Table 7: Feature importance of 265 features. Numbers indicate the region number, described in Supplementary Table 3*

| **All Feature** | **Feature importance** |
| --- | --- |
| AveragePathLength_7 | 0.03901 |
| Degree_84 | 0.03107 |
| GlobalEfficiency_21 | 0.02286 |
| GlobalEfficiency_22 | 0.02115 |
| Degree_163 | 0.02009 |
| LocalEfficiency_22 | 0.01994 |
| Degree_22 | 0.01974 |
| GlobalEfficiency_157 | 0.01938 |
| Degree_85 | 0.01639 |
| LocalEfficiency_67 | 0.01590 |
| GlobalEfficiency_84 | 0.01567 |
| Degree_21 | 0.01538 |
| GlobalEfficiency_83 | 0.01496 |
| Degree_7 | 0.01390 |
| GlobalEfficiency_163 | 0.01381 |
| Degree_129 | 0.01296 |
| GlobalEfficiency_102 | 0.01274 |
| GlobalEfficiency_162 | 0.01245 |
| GlobalEfficiency_129 | 0.01225 |
| Degree_138 | 0.01195 |
| Degree_72 | 0.01175 |
| Degree_81 | 0.01125 |
| GlobalEfficiency_122 | 0.01090 |
| AveragePathLength_158 | 0.01085 |
| Degree_122 | 0.01050 |
| LocalEfficiency_84 | 0.01047 |
| AveragePathLength_163 | 0.00983 |
| Degree_66 | 0.00971 |
| LocalEfficiency_86 | 0.00864 |
| BetweennessCentrality_83 | 0.00857 |
| GlobalEfficiency_104 | 0.00845 |
| Degree_157 | 0.00821 |
| LocalEfficiency_147 | 0.00798 |
| GlobalEfficiency_48 | 0.00796 |
| Degree_83 | 0.00794 |
| AveragePathLength_14 | 0.00786 |
| GlobalEfficiency_164 | 0.00785 |
| GlobalEfficiency_7 | 0.00764 |
| ClusteringCoefficient_22 | 0.00752 |
| AveragePathLength_104 | 0.00736 |
| Degree_65 | 0.00726 |
| Degree_8 | 0.00725 |
| LocalEfficiency_21 | 0.00714 |
| AveragePathLength_162 | 0.00711 |
| AveragePathLength_84 | 0.00687 |
| GlobalEfficiency_158 | 0.00655 |
| AveragePathLength_85 | 0.00635 |
| GlobalEfficiency_137 | 0.00605 |
| GlobalEfficiency_166 | 0.00597 |
| AveragePathLength_33 | 0.00590 |
| AveragePathLength_166 | 0.00579 |
| AveragePathLength_83 | 0.00572 |
| BetweennessCentrality_162 | 0.00560 |
| Degree_104 | 0.00559 |
| LocalEfficiency_150 | 0.00557 |
| BetweennessCentrality_102 | 0.00555 |
| AveragePathLength_65 | 0.00540 |
| LocalEfficiency_33 | 0.00539 |
| AveragePathLength_164 | 0.00528 |
| AveragePathLength_22 | 0.00527 |
| BetweennessCentrality_164 | 0.00523 |
| AveragePathLength_102 | 0.00523 |
| Degree_139 | 0.00510 |
| Degree_101 | 0.00504 |
| GlobalEfficiency_130 | 0.00501 |
| Degree_162 | 0.00481 |
| BetweennessCentrality_145 | 0.00479 |
| Degree_126 | 0.00477 |
| LocalEfficiency_148 | 0.00476 |
| GlobalEfficiency_165 | 0.00475 |
| AveragePathLength_157 | 0.00474 |
| AveragePathLength_71 | 0.00465 |
| Degree_114 | 0.00465 |
| GlobalEfficiency_144 | 0.00455 |
| Degree_165 | 0.00447 |
| Degree_144 | 0.00441 |
| AveragePathLength_21 | 0.00433 |
| ClusteringCoefficient_84 | 0.00432 |
| GlobalEfficiency_81 | 0.00430 |
| GlobalEfficiency_34 | 0.00429 |
| GlobalEfficiency_127 | 0.00427 |
| BetweennessCentrality_85 | 0.00426 |
| Degree_164 | 0.00412 |
| AveragePathLength_42 | 0.00409 |
| GlobalEfficiency_97 | 0.00383 |
| AveragePathLength_147 | 0.00377 |
| ClusteringCoefficient_129 | 0.00374 |
| Degree_36 | 0.00372 |
| BetweennessCentrality_22 | 0.00371 |
| BetweennessCentrality_81 | 0.00362 |
| ClusteringCoefficient_67 | 0.00359 |
| AveragePathLength_61 | 0.00358 |
| AveragePathLength_74 | 0.00348 |
| Degree_96 | 0.00344 |
| LocalEfficiency_85 | 0.00339 |
| BetweennessCentrality_99 | 0.00338 |
| AveragePathLength_41 | 0.00335 |
| GlobalEfficiency_35 | 0.00335 |
| ClusteringCoefficient_20 | 0.00331 |
| GlobalEfficiency_8 | 0.00330 |
| GlobalEfficiency_65 | 0.00325 |
| LocalEfficiency_136 | 0.00319 |
| GlobalEfficiency_63 | 0.00317 |
| ClusteringCoefficient_100 | 0.00316 |
| Degree_41 | 0.00304 |
| LocalEfficiency_35 | 0.00303 |
| ClusteringCoefficient_141 | 0.00281 |
| Degree_71 | 0.00280 |
| LocalEfficiency_88 | 0.00269 |
| BetweennessCentrality_66 | 0.00245 |
| AveragePathLength_148 | 0.00244 |
| Degree_158 | 0.00241 |
| BetweennessCentrality_126 | 0.00240 |
| GlobalEfficiency_33 | 0.00238 |
| Degree_97 | 0.00230 |
| Degree_128 | 0.00228 |
| ClusteringCoefficient_134 | 0.00224 |
| BetweennessCentrality_114 | 0.00221 |
| ClusteringCoefficient_18 | 0.00220 |
| BetweennessCentrality_67 | 0.00216 |
| Degree_130 | 0.00210 |
| ClusteringCoefficient_44 | 0.00209 |
| BetweennessCentrality_34 | 0.00208 |
| ClusteringCoefficient_61 | 0.00199 |
| ClusteringCoefficient_41 | 0.00199 |
| Degree_131 | 0.00198 |
| ClusteringCoefficient_106 | 0.00193 |
| LocalEfficiency_80 | 0.00189 |
| GlobalEfficiency_5 | 0.00188 |
| AveragePathLength_39 | 0.00185 |
| GlobalEfficiency_90 | 0.00182 |
| BetweennessCentrality_158 | 0.00179 |
| BetweennessCentrality_48 | 0.00179 |
| GlobalEfficiency_131 | 0.00176 |
| Degree_48 | 0.00171 |
| Degree_63 | 0.00169 |
| BetweennessCentrality_146 | 0.00167 |
| GlobalEfficiency_12 | 0.00166 |
| ClusteringCoefficient_33 | 0.00164 |
| BetweennessCentrality_153 | 0.00164 |
| AveragePathLength_31 | 0.00161 |
| ClusteringCoefficient_48 | 0.00159 |
| ClusteringCoefficient_125 | 0.00157 |
| LocalEfficiency_5 | 0.00154 |
| ClusteringCoefficient_63 | 0.00154 |
| GlobalEfficiency_74 | 0.00153 |
| LocalEfficiency_158 | 0.00153 |
| LocalEfficiency_34 | 0.00152 |
| AveragePathLength_122 | 0.00149 |
| ClusteringCoefficient_71 | 0.00148 |
| BetweennessCentrality_77 | 0.00146 |
| LocalEfficiency_19 | 0.00146 |
| ClusteringCoefficient_113 | 0.00142 |
| AveragePathLength_129 | 0.00141 |
| AveragePathLength_13 | 0.00140 |
| BetweennessCentrality_88 | 0.00136 |
| LocalEfficiency_130 | 0.00133 |
| LocalEfficiency_18 | 0.00132 |
| ClusteringCoefficient_19 | 0.00132 |
| BetweennessCentrality_163 | 0.00131 |
| ClusteringCoefficient_122 | 0.00129 |
| LocalEfficiency_24 | 0.00129 |
| GlobalEfficiency_138 | 0.00129 |
| BetweennessCentrality_70 | 0.00128 |
| ClusteringCoefficient_23 | 0.00127 |
| ClusteringCoefficient_29 | 0.00127 |
| Degree_23 | 0.00127 |
| ClusteringCoefficient_136 | 0.00125 |
| GlobalEfficiency_103 | 0.00125 |
| AveragePathLength_32 | 0.00118 |
| GlobalEfficiency_39 | 0.00114 |
| AveragePathLength_93 | 0.00112 |
| BetweennessCentrality_154 | 0.00112 |
| ClusteringCoefficient_58 | 0.00110 |
| GlobalEfficiency_136 | 0.00110 |
| AveragePathLength_67 | 0.00101 |
| Degree_49 | 0.00099 |
| BetweennessCentrality_7 | 0.00099 |
| LocalEfficiency_140 | 0.00099 |
| AveragePathLength_57 | 0.00095 |
| AveragePathLength_25 | 0.00093 |
| LocalEfficiency_103 | 0.00092 |
| BetweennessCentrality_28 | 0.00092 |
| GlobalEfficiency_42 | 0.00090 |
| GlobalEfficiency_20 | 0.00090 |
| LocalEfficiency_102 | 0.00087 |
| BetweennessCentrality_20 | 0.00085 |
| ClusteringCoefficient_36 | 0.00085 |
| AveragePathLength_11 | 0.00084 |
| LocalEfficiency_99 | 0.00084 |
| Degree_58 | 0.00083 |
| GlobalEfficiency_67 | 0.00081 |
| ClusteringCoefficient_135 | 0.00081 |
| AveragePathLength_3 | 0.00079 |
| AveragePathLength_27 | 0.00077 |
| AveragePathLength_144 | 0.00074 |
| AveragePathLength_128 | 0.00070 |
| BetweennessCentrality_21 | 0.00070 |
| Degree_146 | 0.00069 |
| ClusteringCoefficient_139 | 0.00056 |
| LocalEfficiency_159 | 0.00055 |
| GlobalEfficiency_110 | 0.00053 |
| AveragePathLength_28 | 0.00051 |
| AveragePathLength_20 | 0.00051 |
| ClusteringCoefficient_142 | 0.00050 |
| LocalEfficiency_141 | 0.00050 |
| AveragePathLength_2 | 0.00049 |
| LocalEfficiency_125 | 0.00049 |
| BetweennessCentrality_84 | 0.00049 |
| Degree_166 | 0.00048 |
| LocalEfficiency_106 | 0.00048 |
| LocalEfficiency_131 | 0.00046 |
| ClusteringCoefficient_4 | 0.00044 |
| GlobalEfficiency_91 | 0.00043 |
| GlobalEfficiency_28 | 0.00043 |
| Degree_30 | 0.00041 |
| BetweennessCentrality_40 | 0.00039 |
| AveragePathLength_81 | 0.00036 |
| GlobalEfficiency_64 | 0.00036 |
| GlobalEfficiency_106 | 0.00035 |
| ClusteringCoefficient_86 | 0.00032 |
| ClusteringCoefficient_39 | 0.00031 |
| AveragePathLength_160 | 0.00023 |
| AveragePathLength_40 | 0.00001 |
| AveragePathLength_48 | 0.00001 |
| AveragePathLength_68 | 0.00001 |
| AveragePathLength_97 | 0.00001 |
| AveragePathLength_109 | 0.00001 |
| AveragePathLength_118 | 0.00001 |
| AveragePathLength_135 | 0.00001 |
| AveragePathLength_154 | 0.00001 |
| BetweennessCentrality_6 | 0.00001 |
| BetweennessCentrality_8 | 0.00001 |
| BetweennessCentrality_17 | 0.00001 |
| BetweennessCentrality_38 | 0.00001 |
| ClusteringCoefficient_5 | 0.00001 |
| ClusteringCoefficient_16 | 0.00001 |
| ClusteringCoefficient_31 | 0.00001 |
| ClusteringCoefficient_43 | 0.00001 |
| ClusteringCoefficient_51 | 0.00001 |
| ClusteringCoefficient_62 | 0.00001 |
| ClusteringCoefficient_65 | 0.00001 |
| ClusteringCoefficient_66 | 0.00001 |
| ClusteringCoefficient_75 | 0.00001 |
| ClusteringCoefficient_145 | 0.00001 |
| ClusteringCoefficient_153 | 0.00001 |
| Degree_37 | 0.00001 |
| Degree_39 | 0.00001 |
| Degree_67 | 0.00001 |
| Degree_160 | 0.00001 |
| GlobalEfficiency_14 | 0.00001 |
| GlobalEfficiency_41 | 0.00001 |
| GlobalEfficiency_53 | 0.00001 |
| GlobalEfficiency_59 | 0.00001 |
| GlobalEfficiency_101 | 0.00001 |
| LocalEfficiency_27 | 0.00001 |
| LocalEfficiency_45 | 0.00001 |
| LocalEfficiency_62 | 0.00001 |
| LocalEfficiency_81 | 0.00001 |
| LocalEfficiency_82 | 0.00001 |
| LocalEfficiency_94 | 0.00001 |
| LocalEfficiency_101 | 0.00001 |
| LocalEfficiency_111 | 0.00001 |
| LocalEfficiency_134 | 0.00001 |
| LocalEfficiency_151 | 0.00001 |

**Reference**

Oliver, I., Hlinka, J., Kopal, J., and Davidsen, J. (2019). Quantifying the Variability in Resting-State Networks. *Entropy* 21, 882. doi: 10.3390/e21090882

PMOD Technologies LLC – PMOD Technologies (n.d.). Available at: https://www.pmod.com/web/ (Accessed October 16, 2022).
